# Green Aspects of Techniques for the Determination of Currently Used Pesticides in Environmental Samples

**DOI:** 10.3390/ijms12117785

**Published:** 2011-11-10

**Authors:** Jolanta Stocka, Maciej Tankiewicz, Marek Biziuk, Jacek Namieśnik

**Affiliations:** Department of Analytical Chemistry, Chemical Faculty, Gdansk University of Technology, Narutowicza Street 11/12, Gdansk 80-233, Poland; E-Mails: maciej.tankiewicz@gmail.com (M.T.); marbiziu@pg.gda.pl (M.B.); chemanal@pg.gda.pl (J.N.)

**Keywords:** pesticides, environment, sustainable development, sample preparation, green analytical chemistry

## Abstract

Pesticides are among the most dangerous environmental pollutants because of their stability, mobility and long-term effects on living organisms. Their presence in the environment is a particular danger. It is therefore crucial to monitor pesticide residues using all available analytical methods. The analysis of environmental samples for the presence of pesticides is very difficult: the processes involved in sample preparation are labor-intensive and time-consuming. To date, it has been standard practice to use large quantities of organic solvents in the sample preparation process; but as these solvents are themselves hazardous, solvent-less and solvent-minimized techniques are becoming popular. The application of Green Chemistry principles to sample preparation is primarily leading to the miniaturization of procedures and the use of solvent-less techniques, and these are discussed in the paper.

## 1. Introduction

Pesticides are a numerous and diverse group of chemical compounds. They make it possible to control the quantities and quality of farm products and food, and they also help to limit diseases in humans transmitted by insects and rodents. They are very widely used not only in agriculture but also in public health, domestic and urban areas, for example as: insect repellents for personal use; rat and other rodent poisons; flea and tick sprays, powders, and pet collars; kitchen, laundry, and bath disinfectants and sanitizers; products that kill mold and mildew; some lawn and garden products, such as weed killers; some swimming pool chemicals [1].

Despite their many merits, pesticides are considered to be some of the most dangerous environmental contaminants because of their ability to accumulate, as well as their mobility and long-term effects on living organisms. The presence of pesticides in the environment is particularly hazardous and their fate and function are still largely unknown. They may cause humans and other living organisms to become more susceptible to diseases [2].

They can also participate in various physical, chemical and biological reactions, as a result of which even more toxic substances may be produced; by accumulating in living organisms, these can lead to irreversible, deleterious changes. The non-rational application of pesticides also adversely affects the environment and humans, increasing susceptibility to diseases and poisoning. Pesticides are a global risk because they move with the wind, rain and sea currents from other regions to places where they have never been used before.

## 2. Currently Used Pesticides

The range of applications of pesticides is continually expanding, hence their consumption is ever increasing and more of them are infiltrating into the environment. In 2009 sales of pesticides in Poland reached 49,760.8 tons, according to figures from the Ministry of Agriculture and Rural Development [3].

It is estimated that EU countries consume more than 300,000 tons of pesticides per annum on crop protection alone. The world market for pesticides is estimated at $33.59 billion, of which the Unites States represents the largest part, in terms of dollars (33%) and pounds of active ingredients (22%) [4]. Table 1 presents the World and U.S. amount of pesticides used in 2006 and 2007 [1].

Currently, more than 800 pesticide active ingredients are present in a wide range of commercial products. These substances belong to more than 100 substance classes. Benzoylureas, carbamates, organophosphorous compounds, pyrethroids, sulfonylureas, or triazines are the most important groups. The chemical and physical properties of pesticides may differ considerably. There are several acidic pesticides; others are neutral or basic. Some compounds contain halogens, others phosphorous, sulfur, or nitrogen. These heteroatoms may have relevance for the detection of pesticides. A number of compounds are very volatile, but several do not evaporate at all. This diversity causes serious problems in the development of a “universal” residue analytical method, which should have the widest scope possible.

The choice of methodology for determining pesticides depends in large measure on the sample matrix and the structure and properties of the target analytes. In view of the numerous legal regulations laying down highest permissible levels of pesticides in various matrices, sensitive and selective analytical techniques are used, appropriate to the low concentrations at which the target analytes occur in them. In addition, each stage in the analytical procedure, as well as this process in its entirety should be validated [5,6].

Traditional methods for the determination of these pollutants are known and described by the EPA—United States Environmental Protection Agency [7] and standards, also Polish [8–12], but often they do not meet expectations, mainly due to the large time and effort required, the need of large amounts of organic solvents and, generally, hazardous and multi-step processes of isolation and enrichment of analytes which could be a source of further contamination and error. Moreover, there is a lack of research devoted to the issue of the new methodologies for the determination of currently used pesticides from different chemical groups. This is mainly due to the fact that these xenobiotics are present in environmental samples at very low concentration levels and the often complex matrix composition, which mandates the use of highly sensitive and selective instrumental techniques, preceded by the isolation and enrichment of analytes. Although reports appear on new analytical procedures for the determination of pesticides, they concern individual chemicals rather than classes of pesticides.

## 3. Green Analytical Chemistry

Due to scientific and public concern about the environment pollution, environmentally-friendly practices have been introduced in different areas of society and research. Green Chemistry is the use of chemistry techniques and methodologies that reduce or eliminate the use or generation of feedstocks, products, by-products, solvents, reagents, *etc*., that are hazardous to human health or the environment [13]. The adverse environmental impact of analytical methodologies has been reduced mainly in three different ways: reduction of the amount of solvents required in sample pre-treatment; reduction in the amount and the toxicity of solvents and reagents employed in the measurement step, especially by automatization and miniaturization; development of alternative direct analytical methodologies not requiring solvents or reagents [14].

The main different steps of the analytical process (sample collection, sample preparation, separation, detection, and data evaluation) make different contributions to environmental pollution and there are different potential ways to make them greener and closer to Green Chemistry principles. The trends in new sample-preparation methods that minimize the amount of reagents and organic solvents contribute to improving the environmentally-friendly features of those methodologies that cannot be applied directly to samples with no sample treatment. Nevertheless, when the use of reagents is unavoidable and their substitution is not feasible, the best alternative is minimization of their consumption. At this point, automation of analytical procedures by means of flow-injection (FI) methodologies plays an important role in the Green Chemistry context [15]. Miniaturization is one way to avoid side effects of analytical methods. In this respect, combination of modern analytical techniques with breakthroughs in microelectronics and miniaturization allows development of powerful analytical devices for effective control of processes and pollution. Combining miniaturization in analytical systems with advances in chemometrics is very important. Of course, development and improvement of new components for instrumentation is critical in Green Analytical Chemistry. Using examples, we have illustrated the power and the versatility of modern analytical systems and their potential for minimizing the consumption of hazardous substances and the amounts of waste generated during assays.

## 4. Green Aspects in Analytical Methodologies

**Figure 1:**
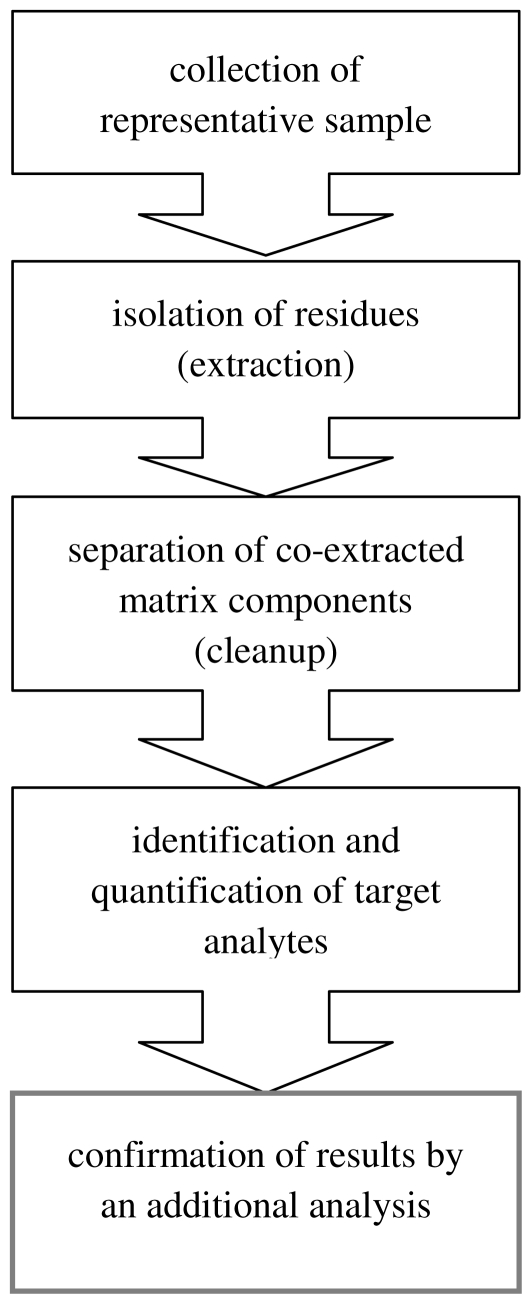
Steps in the determination of pesticide residues in samples characterized by complex composition of the matrix.

Nowadays, the trend is to develop analytical methods enabling a broad spectrum of analytes to be determined in a single analytical run (multiresidue methods—MRM); but the problem here is that the compounds to be determined simultaneously, often present at low concentrations, have different physicochemical properties depending on their chemical structure [12]. Figure 1 presents the steps of a multiresidue method. Such a methodology, apart from being able to determine a large number of compounds in one run, should:

- ensure maximum removal of interferents from extracts,
- give large recoveries of target compounds, high sensitivity and good precision,
- be environmentally-friendly, *i.e*., require the smallest possible quantities of samples and chemical reagents, especially organic solvents,
- be cheap, quick and easy to carry out.

Generally the analytical procedure consists of numerous stages, the most important of which is the collection of a sample and its preparation for analysis. This stage is a complicated process, and its operations can be both a cause of analyte loss and a source of additional contamination. All errors at this stage will affect the final result of determination. A further difficulty is the fact that the collection and preparation of a sample takes up to *ca.* two thirds of the time required to perform the complete analysis. New techniques have been developed which eliminate many of these inconveniences and also increase the precision, throughput, reproducibility, and cost-effectiveness. In many cases the capability for smaller initial sample sizes, even for trace analyses, is also essential [16].

**Figure 2:**
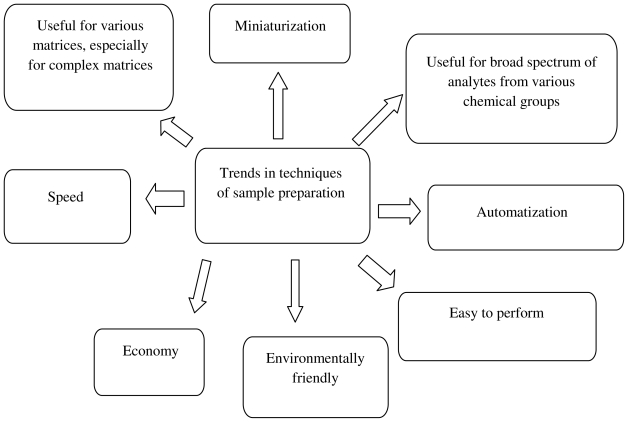
Trends in the development procedures for determination of trace constituents in samples characterized by complex composition of the matrix.

From an analytical point of view, environmental and food samples are highly diverse and complex: the factors affecting the nature of the sample are the sampling site, the type of matrix, the presence of interferents and the low concentration of target analytes. Whether or not the analysis yields reliable information about the sample content depends to a large extent on the proper sample preparation. The quality of sampling and sample pretreatment largely determine the success of an analysis from complex matrices. Ideally, sample preparation should be as simple as possible, because it not only reduces the time required, but also decreases the possibility of introducing contaminants. Figure 2 presents trends in the development of techniques of sample preparation.

One of the oldest extraction techniques, and at the same time one of the most common in routine sample preparation, is liquid-liquid extraction (LLE). The solvents in LLE are usually dichloromethane [17–20], mixtures of petroleum ether and dichloromethane [21] or hexane and dichloromethane [18]. LLE is recognized as an attractive technique for screening tests of unknown pesticides [22,23] not only because of its simplicity, efficiency, minimal operator training, but also because of its wide acceptance in many standard methods. However, this technique has a number of drawbacks: it requires relatively large quantities of toxic solvents and multistage operation, there is a risk of emulsion forming during agitation, and there is the problem of disposal of the post-extraction solvents. To achieve the desired preconcentration coefficient, the excess solvent usually has to be evaporated. Also extract cleanup is often necessary. To minimize these disadvantages, numerous improvements have been made to this method, most of which have involved miniaturizing the process to reduce the amounts of solvents consumed.

Microextraction techniques, such as: liquid-liquid microextraction, dispersive liquid-liquid microextraction, single drop microextraction, solid-phase microextraction (SPME), stir-bar sorptive extraction (SBSE), liquid-phase microextraction (LPME), and on-line solid-phase extraction (SPE), have several advantages over the traditional approaches of liquid–liquid extraction (LLE) and conventional SPE [24–28].

The main advantages are minimal consumption of harmful solvents, and typically, the high enrichment factor. The improved sensitivity makes it possible to electron the amount of sample needed in the analysis. All these techniques are readily combined with GC, either off-line, at-line or sometimes even on-line [29]. Off-line procedures are good alternative when the number of samples is small, because there is usually no need for an automated method and the time-consuming development of such a method. Conventional methods will suffice. Setting up an automated method, either at-line or on-line, becomes more worthwhile when the number of analyzed samples increases. Automation typically improves the quality of the data, increases the sample throughput, decreases costs and improves the productivity of personnel and instruments. On-line systems are beneficial when the analytes are labile, the amount of sample is limited, or very high sensitivity is required. The selection of an extraction technique is made on the basis of several factors. Naturally, the sample preparation must be tailored to the final analysis. The sample matrix and the type and amount of analytes in the sample are of primary importance. Also crucial are speed of extraction, complexity of the instrumentation, simplicity and flexibility of the method development, and ruggedness of the method. Moreover, a method good for target-compound analysis may not be good for comprehensive chemical profiling of samples. Selectivity of the sample preparation is often a key factor for target-compound analysis while an exhaustive extraction is the better choice for profiling.

In practice, these novel developed techniques can be performed by following two general methodologies. These are solvent microextraction, where the extraction is performed by using a small amount (drop) of water-immiscible solvent suspended in a sample (MLLE, SDME, HS-SDME, CFME) and extraction via a membrane (HF(2)ME, SLME, MMLLE, MASE) which can be a selective barrier between two phases (see Table 2). The dispersion of very fine droplets of organic solvents into the aqueous phase in a ternary solvent component system (liquid samples) is another new option and called dispersive liquid-liquid microextraction (DLLME) [30,31]. Table 2 presents the most commonly used novel techniques for sample preparation in pesticides analysis.

These microextraction techniques eliminate the disadvantages of traditionally used extraction methods such as time-consuming operation and need for specialized apparatus. They are inexpensive and offer considerable freedom in selecting appropriate solvents for the extraction of different analytes. Moreover, they minimize exposure to toxic organic solvents.

Recently also increasing interest is observed in ad/absorption-based methods using beds of solid enrichment sorbents, which have gradually replaced conventional LLE for sample pretreatment and have gained wide acceptance because of their simplicity and economy in terms of time and solvent needs. Sorptive extraction techniques mainly include solid-phase extraction (SPE), solid-phase microextraction (SPME) and stir bar sorptive extraction (SBSE) (Table 3).

**Figure 3:**
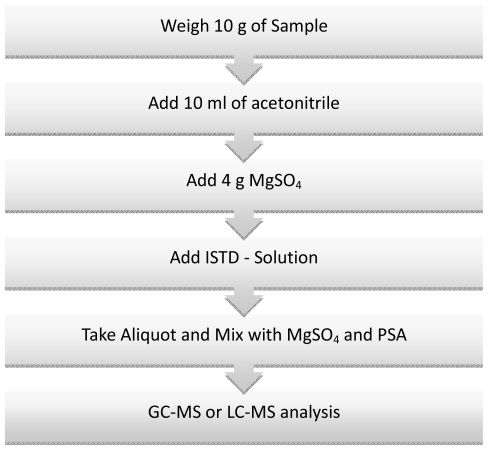
Steps in the QuEChERS procedure of sample preparation for the determination of pesticide residue in fruit and vegetables.

Approaches are being sought to develop pesticide determination techniques that are quick, easy, cheap, effective, rugged and **s**afe. **QuEChERS** is a highly effective sample preparation technique for pesticide residue analysis. It is a combination of liquid-liquid extraction (LLE) and solid-phase extraction (SPE) and was developed by Anastassiades *et al*. [72]. The original Quechers method is based on a number of stages (see Figure 3). Samples are milled in frozen state (dry ice is added) to get the best recovery. Extraction is done in acetonitrile buffered at pH 5–5.5. After centrifuged, the organic phase is cleaned-up by dispersive SPE using primary secondary amine—PSA (and graphitized carbon black—GCB as necessary). Additional MgSO_4_ is added to remove any residual water. The PSA treated extract is acidified with formic acid to improve the stability of base-sensitive pesticides. The extract is ready for GC and LC analysis. For samples with low water content (<80%), water is added before the initial extraction to get a total of *ca*. 10 mL water. Quality control is performed by adding ISTD to the acetonitrile extraction step.

The consumption of sample and toxic solvents with the QuEChERS method is minimal. By applying QuEChERS to the determination of pesticides in fruit and vegetables, matrix effects are eliminated and high recoveries of target analytes are possible. The method can be modified depending on the type of sample and the target analytes. To improve the extraction of polar organophosphorus pesticides, the method is modified by the addition of acetic acid. When samples of citrus fruit are under investigation, protective wax coatings can be removed by freezing the samples for at least one hour. For samples, with a high content of carotenoides or chlorophyll, cleanup with PSA is not satisfying and there is a need to use GCB which is best in handling and effect. QuEChERS approach takes advantages of the wide analytical scope and high degree of selectivity and sensitivity provided by gas and liquid chromatography (GC and LC) coupled to mass spectrometry (MS) for detection. QuEChERS is a multi-residue method with fast sample preparation and low solvent consumption [73–79].

Nguyen *et al.* proposed a multiresidue method based on the QuEChERS sample preparation method and gas chromatography with the electron impact mass spectrometric detection in the selected ion monitoring mode (GC-SIM-MS) for the routine analysis of 107 pesticides in cabbage and radish. The recoveries for all the pesticides were from 80% to 115% with relative standard deviation lower than 15%. The limits of quantifications were in the range 0.002–0.05 mg/kg [80].

The analysis of pesticides poses special problems for the analysts, since the pesticides belong to different groups of chemical substances, having a broad range of polarity and acidic/base characteristic. Pesticide analysis methodologies (usually in ultratraces range-μg L^−1^) require typically analytical separative techniques such as gas chromatography (GC) or liquid chromatography (LC) which can be associated with a wide variety of selective detection methods:

➢ ECD (Electron Capture Detector)—highly sensitive in relation to compounds containing electronegative atoms,
➢ FPD (Flame Photometric Detector)—applied in the determination of organophosphorus compounds,
➢ NPD (Nitrogen Phosphorus Detector)—used for the simultaneous determination of organonitrogen and -phosphorus pesticides.

Most pesticides are volatile and thermally stable, and therefore are amenable to GC. In contrast to GC, procedures based on application of LC technique have the advantage of being suitable for thermally unstable and polar/ionic pesticides, as these compounds require derivatization prior to GC analysis. The selective detectors are the most common used in routine residue analysis. Unfortunately, these do not allow confirmation of the analysis results without ambiguity [81]. The detection by mass spectrometry (MS) employing quadrupole, ion trap and/or time-of-flight analyzers offers simultaneously the confirmation and the quantification of numerous pesticides [82]. It has become very popular in laboratories performing monitoring of pesticide residues analysis [83]. As powerful as MS is, the low-resolution, scanning MS system has limits in data collection rate, avoidance of interferences, and spectral information provided for identification purposes. Currently, low-resolution (unit mass) MS detectors employing either single quadrupole or ion trap analyzers are most routinely used in applications [84–86]. Furthermore, innovations in chromatographic particle chemistry (from 5 to 3.5 or 1.8 μm packing in LC, as well as new bonding chemistries) have improved the separation of pesticides [87].

The confirmation of analysis results can also be performed by another independent method. The conditions of the process can be altered, by changing the temperature program or by using a different chromatographic column. It is crucial to obtain confirmation by another method as identification based solely on retention times is insufficient. Tandem mass spectrometry (MS/MS) improves sensitivity and selectivity of analytical methods. In this technique, ions that were separated in the first analyzer are again fragmented and the derivative ions analyzed in the second one. The chromatogram background is reduced, as a result of which the signal value is enhanced with respect to noise and the LOD (limit of detection) of the target analytes is lowered [88,89]. Better chromatographic peak resolution and a smaller influence of the matrix on the final result can also be achieved using two-dimensional (2D) gas chromatography (GCxGC). This uses two columns: the partially separated constituents from the first column are further separated in the second one by a different mechanism. The advantage of this method is that the separation mechanisms in the two columns are independent of each other, so that constituents that were co-eluted from the first column can be separated. Moreover, GCxGC can simplify the preparation of samples for determination of the presence of pesticides. This method is widely used because of its high resolving power, greater sensitivity and ordered nature of the chromatograms.

Fast GC is equally frequently used to shorten the time of analysis, which allows increasing sample throughput, and to obtain better peak resolution. Consequently the laboratory operating costs per sample can be reduced significantly [90,91]. Compared to classical GC, it requires shorter capillary columns with a smaller diameter and thinner film of stationary phase *ca*. 0.1 μm in thickness, as well as a faster flow rate and higher pressure of the carrier gas. These parameters yield determination results in higher precision [88,92,93].

Alder *et al*. applied multiresidue GC-MS method with electron impact ionization (EI) and the combination of LC with tandem mass spectrometers (LC-MS/MS) with electrospray ionization (ESI) for determination of 500 high priority pesticides. Only for one substance class, the organochlorine pesticides, GC-MS achieves better performance. For all other classes of pesticides a wider scope and better sensitivity were observed for LC-MS/MS. Table 4 lists the number of pesticides in each class that could not be detected by either of the two methods (GC-MS and LC-MS/MS).

Based on the data from Table 2 it can be concluded, that more pesticides and their metabolites can be determined by using LC and ESI than by GC–MS. It is well known that sulfonyl or benzoyl ureas and many carbamates or triazines can be better or exclusively detected by LC–MS/MS techniques. Furthermore, a wider scope of LC–MS/MS was found for most of the other chemical classes too, for example, the organophosphorus pesticides. Only 49 compounds out of 500 exhibited no response, if LC–MS/MS in combination with positive and negative ESI was used. On the other hand, 135 pesticides/metabolites could not be analyzed by GC/MS using EI ionization, most often because of incompatibility with evaporation of the intact molecule in the GC injector. Both of these instruments have special merits, but neither of them can detect the full range of all pesticides. However, if the selection of the most appropriate techniques is focused on the enforcement of maximum residue levels, simultaneous identification, and quantification of a very large number of target analytes will be more important than the detection, identification, and quantification of non-regulated (non-target) pesticides and/or metabolites [94].

Research is continuing into the improvement of existing analytical methods and the development of new ones capable of supplying reliable results for a wide range of analytes in a short time and will be more economical and environmentally friendly.

## 5. Conclusions

Due to scientific and public concern about environment pollution, environmentally-friendly practices have been introduced in different areas of society and research. Investigation of green analytical methodologies encompasses a number of strategies to minimize or to eliminate the use of toxic substances and the generation of waste. The main focus has been on the development of new routes to minimize the amounts of side products and to replace toxic solvents. Progress in analytical methodologies has contributed to the development of new, greener options.

## Figures and Tables

**Table 1:** The World and U.S. amount of pesticides used in 2006 and 2007 (in millions of pounds).

Type of pesticide	World market	US market	US percentage of world market [%]
**2006**			

herbicides	2018	498	25
insecticides	955	99	10
fungicides	519	73	14
other	1705	457	27
total	5197	1127	22

**2007**			

herbicides	2096	531	25
insecticides	892	93	10
fungicides	518	70	14
other	1705	439	26
total	5211	1133	22

**Table 2:** The most commonly used novel (green) techniques for sample preparation in pesticide analysis.

Technique of Sample Preparation	Volume of Organic Solvent	Description	Literature
MLLE (micro liquid-liquid extraction)	about 1 mL per 1 L of sample	It is possible to decrease the consumption of organic solvents by miniaturization and proper design of extraction vessel. The most commonly used solvents for microextraction are dichloromethane, toluene and methyl-tert-butyl ether.	[32,33]
SDME (single drop microextraction)	0.9–1.5 μL	The extraction phase is a drop of organic solvent (e.g., n-hexane, toluene, butyl acetate) suspended at the tip of microsyringe, so it is practically a solvent-free method. It can be carried out in two different ways by direct immersion (DI) or from the headspace (HS). Analyte isolation and preconcentration take place in a single step. The extraction process is assisted by mixing. When the extraction is complete, the microdroplet is directly injected into a gas chromatograph (GC) or high-performance liquid chromatograph (HPLC) for further analysis. The universality of SDME makes it widely applicable to the analysis of pesticides in samples with a complex composition containing target analytes in trace amounts.	[34–37]
CFME (continuousflow microextraction)	1–5 μL	This technique is similar to SDME. The drop of extraction solvent is injected by microsyringe into a glass chamber (0.5 mL) and held at the outlet tip of a polyetheretherketone (PEEK) connecting tube. The sample solution flows past the tube and through the glass extraction unit to waste. Extraction takes place continuously between the organic drop and the flowing sample solution. Because the drop of solvent makes full contact with the sample solution, the technique achieves higher concentration factor than static SDME.	[30]
DLLME (dispersive liquid-liquid microextraction)	disperser solvent 0.5–2 mL; extraction solvent 10–50 μL	The mixture of extraction solvent (e.g., chlorobenzene, carbon tetrachloride, tetrachloroethylene, carbon disulfide) and disperser solvent (e.g., acetone or methanol) is rapidly injected into an aqueous sample, resulting in the formation of a cloudy solution. The DLLME procedure is very convenient to operate and extraction could be completed in a few seconds. DLLME has advantages of simplicity of operation, rapidity and low cost. DLLME can be coupled with GC and HPLC. The non-selective characteristic of the extraction solvents can be sometimes a disadvantage. Recently He *et al*. used as extraction solvent ionic liquid 1-octyl-3- methylimidazolium hexafluorophosphate ([C_8_MIM][PF_6_]) for the determination of organophosphorus pesticides in water sample. Ionic liquids belong to non-molecular solvents with unique properties such as negligible vapor pressure associated to a high thermal stability. Hydrophobic ionic liquids incorporating the imidazolium cation and hexafluorophosphate anion have higher density than water. Compared with commonly used solvents they are more compatible with reversed-phase HPLC due to the non-harmfulness to column.	[38–45]
HF(2)ME (hollow fiber-protected two-phase solvent microextraction)	2–3 μL	The method is straightforward, quick, inexpensive and eliminates necessity of extract cleanup prior to final determination. Toluene, hexane or 1-octanol are usually used for the extraction of pesticides. It is based on the partition of analytes between the aqueous solution and the small quantity of organic solvent in a microporous tube (the rod configuration). The hollow fiber can be also in the U-shape configuration. The process is assisted by stirring. About 1–1.5 μL of extract is taken for further analysis using appropriate chromatographic techniques. For more complex matrices and moderately polar pesticides. Basheer *et al.* developed binary solvent based on HF(2)ME with GC-MS. The mixture (1:1) toluene: hexane was used as solvent. The limits of detection (LODs) were in the range of 0.3–11.4 ng L^−1^ and relative standard deviations (RSD) were 9–13%. This technique gave higher analytes enrichment, especially when applied to complex matrices (wastewater).	[40,46]
LPME-SFO (liquid-phase microextraction based on the solidification of a floating organic drop)	10 μL	The small volume of an extraction solvent (usually 1-undecanol) is floated on the surface of aqueous solution. The process is assisted by stirring. After the extraction, the floated extractant droplet can be collected easily by solidifying it at low temperature. The solidified organic solvent can be melted quickly at room temperature, which is then determined by either chromatographic or spectrometric methods. The technique is cheap, quick and sensitive, but the rate of extraction is slightly slow.	[47,48]
MMLLE (microporous membrane liquid-liquid extraction)	0.2 mL	Advantages of this technique compared to LLE are small sample volumes, the lack of emulsion formation, the clean extracts obtained and it can be coupled online to gas chromatography. The flat-sheet membrane extraction unit consisted of two blocks, one made of poly(tetrafluoroethylene) (PTFE) and the other of poly(etheretherketone) (PEEK). The membrane constitutes a barrier between two phases: acceptor (usually toluene) and the aqueous donor solution (sample). The donor solution is pumped to the donor channel of the membrane block, while the acceptor is stagnant during the extraction period.	[49,50]
LLSME (liquid-liquidsolid microextraction)	6–100 μL	This technique combines the advantages of solid-phase microextraction and liquid-phase microextraction. The molecularly imprinted polymer (MIP)—coated silica fiber is protected with a length of porous polypropylene hollow fiber membrane which is filled with water-immiscible organic phase (usually toluene). This technique is a three-phase microextraction approach. It is fast, selective and sensitive method for trace analysis of pesticides in complex aqueous samples.	[51,52]

**Table 3:** The most commonly used novel techniques for sample preparation in pesticide analysis (minimization of toxic reagents).

Technique of Sample Preparation	Volume of Organic Solvent	Description	Literature
SPE (solid-phase extraction)	<15 mL	The advantages of this method are: requires a lower volume of solvent than traditional LLE, involves simple manipulations which are not time consuming, the SPE cartridges can be used for short-term storage of the species and provides high enhancement factors proportional to the volume of water passed through the SPE cartridge. Conventional sorbents such as C_18_ silica, graphitized carbon black and macroporous polystyrene divinylbenzene (PS-DVB), show low retention for polar compounds. In order to improve the extraction efficiency for polar compounds, the development of new adsorbents and modification of the adsorbents by introducing the polar groups become a major research direction. Nanomaterials are one kind of novel adsorbents. Carbon nanotubes (CNTs), including single-walled carbon nanotubes (SWCNTs) and multi-walled carbon nanotubes (MWCNTs), are a kind of carbonaceous nanomaterial and have received significant attention in many fields. In recent years, molecular imprinting polymer (MIP) technology with high selectivity evolves rapidly. MIP technology is now well established for the preparation of tailor-made polymers with cavities capable to extract or clean-up of OPPs.	[53–61]
SPME (solid phase microextraction)	solvent-free extraction	This technique uses polymer-coated fibers to extract analytes from aqueous or gaseous samples. After extraction, the analytes are either desorbed thermally by exposing the fiber in the injection port of a GC or chemically desorbed and analyzed by LC. SPME does not require the use of organic solvents. It is quick, universal, sensitive and convenient for use in the field and is simply applied in sample preparation. However the fiber is comparatively expensive, fragile and has limited lifetime. The materials used for coating fibers include: polydimethylsiloxane (PDMS), polyacrylate (PA), and also mixtures of: polydimethylsiloxane and polydivinylbenzene (PDMS-DVB), carbowax and polydivinylbenzene (CW-DVB), carbowax and molecularly imprinted resin (CW-TP). Depending on where the fiber is situated in relation to the sample, SPME can be carried out in two different ways by direct immersion (DI) or from the headspace (HS). The advantage of this method is that the limited capacity of the adsorbent precludes column overloading.	[62–68]
SBSE (stir bar sorptive extraction)	solvent-free extraction	This techniques uses a 1.5 cm long glass magnetic stirrer coated with a thick layer of polydimethylsiloxane (PDMS) where sorption usually takes place. Its sorption capacity is a hundred times greater in comparison with sorption capacity of SPME fibers. Its main advantage is high sensitivity and a wide application range that includes volatile aromatics, halogenated solvents, polycyclic aromatic hydrocarbons, polychlorinated biphenyls, pesticides or organotion compounds. Because of the non-polar character of PDMS, the SBSE cannot be used to extract strong polar compounds unless derivatization was utilized.	[69–71]

**Table 4:** Pesticide classes and number of pesticides in each class that cannot be detected by GC-MS or LC-MS/MS.

Chemical Class	Number of Pesticides in That Class	Not Detected by GC-MS	Not Detected by LC-MS/MS
organophosphorus	81	0	1
carbamate	43	17	1
organochlorine	40	0	33
sulfonylurea	26	26	0
triazole	24	1	0
triazine	23	6	0
urea	22	16	0
pyrethroid	19	0	2
aryloxyphenoxy-propionate	12	4	0
aryloxyalkanoic acid	10	9	0
other	200	56	12
Total number	500	135	49

## Acronyms

CFME: Continuous-Flow Microextraction
CW: Carbowax
DAD: Diode Array Detector
DI: Direct Immersion
DLLME: Dispersive Liquid-Liquid Microextraction
DVB: Polydivinylbenzene
ECD: Elektron Capture Detector
EI: Elektron Impact Ionization
ESI: Electrospray Ionization
FI: Flow-Injection
FPD: Flame Photometric Detector
GC: Gas Chromatography
GCB: Grapfized Karbon Black
GCxGC: Two-Dimensional Gas Chromatography
HF(2)ME: Hollow Fiber-protected two-phase Solvent Microextraction
HPLC: High Performance Liquid Chromatography
HS: Head Space
ISTD: Two Different Internal Standards
LLE: Liquid-Liquid Extraction
LOD: Limit of Detection
LPME: Liquid- Phase Microextraction
MASE: Membrane-Assisted Solvent Extraction
MLLE: Micro Liquid-Liquid Extraction
MMLLE: Membrane Micro Liquid-Liquid Extraction
MRM: Multiresidue Methods
MS: Mass Spectrometry
MS/MS: Tandem Mass Spectometry
NPD: Nitrogen-Phosphorus Detector
PDMS: Polydimethylsiloxane
PSA: Primary Secondary Amine
SBSE: Stir Bar Sorptive Extraction
SDME: Single-Drop Microextraction
SPE: Solid-Phase Extraction
SPME: Solid Phase Microextraction
TSD: Thermionic Specific Detector
UV: Ultra-Violet
